# Understanding Measurement in Light of Its Origins

**DOI:** 10.3389/fpsyg.2013.00113

**Published:** 2013-03-20

**Authors:** Stephen Humphry

**Affiliations:** ^1^Faculty of Education, The University of Western AustraliaPerth, WA, Australia

**Keywords:** measurement, ratio, proportion, classical theory, representational theory, psychometrics, metrology

## Abstract

During the course of history, the natural sciences have seen the development of increasingly convenient short-hand symbolic devices for denoting physical quantities. These devices ultimately took the form of physical algebra. However, the convenience of algebra arguably came at a cost – a loss of the clarity of direct insights by Euclid, Galileo, and Newton into natural quantitative relations. Physical algebra is frequently interpreted as ordinary algebra; i.e., it is interpreted as though symbols denote (a) numbers and operations on numbers, as opposed to (b) physical quantities and quantitative relations. The paper revisits the way in which Newton understood and expressed physical definitions and laws. Accordingly, it reviews a compact form of notation that has been used to denote both: (a) ratios of physical quantities; and (b) compound ratios, involving two or more kinds of quantity. The purpose is to show that it is consistent with historical developments to regard physical algebra as a device for denoting relations among ratios. Understood in the historical context, the objective of measurement is to establish that a physical quantity stands in a specific ratio to another quantity of the same kind. To clarify the meaning of measurement in terms of the historical origins of physics carries basic implications for the way in which measurement is understood and approached. Possible implications for the social sciences are considered.

According to definitions of measurement that have been adopted in the social sciences, measurement involves the association or assignment of numbers to entities. Definitions of this kind are representational in nature. Perhaps the most influential is the definition stated by Stevens (1946, p. 677): “measurement, in the broadest sense, is defined as the assignment of numerals to objects or events according to rules.” Michell (1999, pp. 15–19) has provided a compelling argument that, despite being incompatible with the traditional conception of measurement in the natural sciences, Stevens’ definition has been the model for many psychologists. Accordingly, representational definitions have also been stated in Item Response Theory, perhaps most notably by Lord and Novick (1968).

The most elaborate attempt to connect measurement in the social sciences to measurement in physics is found in the *Foundations of Measurement* (Krantz et al., 1971) and preceding work. The authors attempted to set out an axiomatic basis for measurement that involves so-called representation theorems. In Volume 1 of the *Foundations of Measurement*, Krantz et al. (1971, p. 1) characterize measurement as follows: “When measuring some attribute of a class of objects or events, we associate numbers … with the objects in such a way that the properties of the attribute are faithfully represented as numerical properties.” The *Foundations* essentially takes the use of convenient short-hand symbolic devices to an extreme whereby the *symbols* of physical algebra are interpreted as though they refer only to numbers and their operations. The axioms of the representational theory refer either to qualitative relations or numerical properties, but not directly to quantitative attributes. In this way, the formal aspect of the theory is kept distinct from reference to qualitative empirical content. Consistent with this, Michell (1999, p. 208) has said of the representational school: “For the most part, Suppes, Luce, and their associates avoided (this concept of quantity) in treating measurement.” Kyburg (1996) and Berka (1983) put forward related observations and criticisms of the representational theory.

In contrast with representational theory, Michell has brought attention to what he refers to as the classical theory of measurement: “scientific measurement is properly defined as the estimation or discovery of the ratio of some magnitude of a quantitative attribute to a unit of the same attribute” (Michell, 1997, p. 358). In this definition, quantitative attributes are given an ontological status and a ratio between magnitudes of quantities is taken to be a real number.

This classical theory is frequently assumed in physics and metrology. In metrology, the Bureau of Weights and Measures (BIPM) claims a mandate to maintain world-wide uniformity of measurements and their traceability to the International System of units (SI). The BIPM implicitly adopts the classical definition in its definition of a unit: “Thus, a *measurement unit* is a scalar quantity, defined and adopted by convention, with which any other quantity of the same kind can be compared to express the ratio of the two quantities as a number” [Bureau International des Poids et Mesures (BIPM), 2006, p. 24].

Galileo, Newton, and other pioneers expressed physical relations as proportionalities among ratios of physical quantities. As will be seen, the classical theory of measurement is more closely connected with the origins of physics than is the representational theory by virtue of its reference to the concept of ratio.

This paper is structured as follows. It begins with a focus on the genesis of physics and, in particular, the Greek-inspired tradition of understanding physical relations in terms of proportion and ratio. It then provides an overview of increasingly convenient abbreviations that were used for proportionality statements, which led to a subsequent divergence of thought in the form of the classical and representational views of measurement. It is argued that the use of increasingly abstract and abbreviated statements came at a cost to the directness of insights into natural relations. Next, a compact form of notation is used to denote ratios, compound ratios, and proportionalities among ratios; and this notation is employed to show a parallel between algebra and proportionality statements. The concept of a compound ratio is then explained and it is shown that although compound ratios have a formal similarity with multiplication, they have a distinct conceptual basis. Lastly, the most basic implications for measurement in physics are explained, and implications for the social sciences are briefly considered.

## Proportion and Ratio in Geometry and Classical Physics

Historically, the pioneers of classical physics, from Galileo to Newton and Faraday, expressed physical relations as the proportionality of ratios of two or more kinds of quantities. Newton made extensive use of geometrical lines to denote quantities such as forces (Newton, 1846; Roche, 1998). In the *Principia*, he repeatedly referred to quantities as being either proportional or inversely proportional to one another in dynamical physical situations. Thus, Newton said:

If in comparing indetermined quantities of different sorts one with another, any one is said to be as any other directly or inversely, the meaning is, that the former is augmented or diminished in the same ratio with the latter, or with its reciprocal (1846, p. 100).

As an example, if a force f is augmented to become f′, and the resulting acceleration of a body a is augmented to become a′, then f: f′ may be in the same ratio as a: a′. Newton here employs Euclid’s use of “in the same ratio” to describe proportionality. Coulomb and Faraday also referred in this way to quantities as being proportional or inversely proportional to one another (Silsbee, 1962).

### Symbols for numbers in physico-mathematics

Proportion and ratio were central to Euclid’s *Elements*. In the Greek-inspired tradition of proportion and ratio, ratios were not conceived as being literally equal to numbers. Rather “[r]atio belonged … to the category of relation and was not a simple quantity or a single number” (Roche, 1998, p. 46). Ratios were therefore not even conceived as being literally *equal* to one another. In his historical analysis of Euclid, Grattan-Guinness (1996) observes: “While he speaks of the equality of numbers and of magnitudes, Euclid never says that ratios are “equal” to each other, only that they are “in the same ratio”, or that one ratio “is as” the other in a proportion proposition.

Not surprisingly, therefore, the idea that a ratio could be denoted by a single number – referred to as either its exponent or denomination – met with considerable resistance at a certain time in history. In the long run, however, “[t]he slowly growing claim to represent all ratios by numbers gradually brought about a merging of the concept of ratio as a single number” (Roche, 1998, p. 76). This merged concept is evident in the classical theory (or definition) of measurement.

Because representational definitions predominate in the social sciences, it is instructive to understand developments in physico-mathematics that allowed these definitions to emerge. The route from the origins of physics to the modern-day representational conception appears to be roughly as follows. First, physical relations were expressed, in words and with geometric analogies, as ratios and compound ratios. Newton followed in this tradition and employed the notational identification of lines with physical quantities, such that the lengths and directions of lines denoted the magnitudes and directions of the quantities. Roche (1998, p. 89) summarizes this and ensuing historical development as follows.

This was not a reduction of the physical to a line … But it was the notational identification of the two concepts. It was extremely convenient, of course, since all of the operations of geometrical algebra could now be applied to these concepts without the restrictions imposed by a proportionality symbolism which referred directly to the natural quantity. This identification eventually passed from lines to numbers and even to the formal symbol itself which stood for the physical quantity.

Later, Laplace interpreted symbols in physical equations as representing “numbers abstracted from well-defined procedures of measurement” (Roche, 1998, p. 138). This was a critical departure because once numbers were seen as abstracted and independent of the empirical world, it became possible to construe the purpose of measurement as being to represent empirical entities with numbers; i.e., it became possible for the representational view to emerge. Michell (1993) details later developments and argues that Russell’s (1903) was the first explicitly representational theory of measurement. Representational theorists themselves provide a largely different account with an emphasis on events that occurred in the late eighteenth and early nineteenth centuries (Diez, 1997; Luce and Suppes, 2002).

### Divergence of views of measurement

Roche (1998, p. 138) argues that the view that only “abstract numbers can appear in the algebra of physics reflects, perhaps, a response to the classical tradition of dealing with physical quantities itself rather than its measure, and the need to distance the algebra of physics from the manipulations of such quantities.” It is perhaps not surprising then that there was subsequently a divergence in the way in which the abstracted numbers were interpreted, which continues today. In the classical view, as defined by Michell (1993), ratios are numbers. This view preserves a key aspect of the Greek tradition but incorporates the use of an abbreviation, as explained to follow. In the representational view there is no connection to ratios, as summarized in the following excerpt.

Essentially, then, there are two features dividing the classical and representational theories of measurement: the role of ratios of quantities and the place of numbers. According to the classical theory, these two are logically connected: ratios of quantities are numbers, and this fact is the basis of measurement. According to the representational theory, numbers do not derive from ratios of quantities. They are quite independent of them and the place of numbers in measurement is determined by the structural similarity between qualitative and quantitative systems. Hence, according to the representational theory, numbers are assigned to empirical entities in measurement. According to the classical theory, numbers are discovered as relations between empirical entities in measurement (Michell, 1993, p. 190).

Despite the emergence of the representational view, the classical view continues to exist in physics. Why did the abstract algebraic view not supplant the classical one entirely? It would be somewhat surprising if proportionality statements used in the conception of physics were less apt to describe natural relations than algebra, which was applied later. It seems more likely that the *convenience* of short-hand devices was the principal reason for their increasing usage. It has been argued, though, that the convenience of physical algebra came at the cost of explicit attention to key features of physical relations, as characterized in the following excerpt.

Several key features of this Greek-inspired tradition … deserve careful attention. Pairs of like magnitudes are compared with other pairs which are often physically different, such as pairs of weights with pairs of distances in the theory of the balance. This is very different from the modern algebraic language of physics which compares single values of different quantities. Furthermore, a proportionality statement seems to be a little closer to describing the natural relation than does the language of algebra (Roche, 1998, p. 47).

The two lines of thought continue to be evident today such that the algebra and arithmetic of physics seem to be “interpreted formally in some contexts and physically in others” (Roche, 1998, p. 222).

## Reconciling Modern Physical Notation with Its Origins

How, then, might we reconcile the modern reference to the equality of ratios and numbers in physics with its origins? Krantz et al. (1971, p. 459) observed: “Most texts on dimensional analysis appear to take (physical) algebra for granted, and they do not attempt to formulate explicitly what is involved.” While this is true enough, by using appropriate notation it is possible to explicitly formulate proportionality statements in a way that shows a clear and direct parallel with modern-day algebraic notation.

Newton’s statement of the second law of motion is used to illustrate this parallel. Newton (1846, p. 83) stated the second law as follows: “The alteration of motion is ever proportional to the motive force impressed; and is made in the direction of the right line in which the force is impressed.” Here, motion refers to what is now commonly referred to as momentum. We may express Newton’s second law as a formal proportionality statement, as follows:

(1) $${\text{f}}^{\prime }:\text{f}::{\text{p}}^{\prime }:\text{p},$$

where f and p refer to the force impressed upon a body at a given moment in time and the resulting momentum of the body, and f′ and p′ refer to the force impressed at another moment on the same body and its resulting momentum, respectively. In statement (1) “::” means “is as” or “is proportional to.” The alteration of momentum is therefore expressed by the ratio p′: p and is proportional to the alteration to the motive force, f′: f. The notation “::” was used by Wallis (1685). Given the objectives of the paper, it ought to be noted that statement (1) is already an abbreviated form of a proportionality statement that omits reference to key elements of the full statement of the law by Newton in words, such as the nature of causality through reference to a force impressed upon a body.

As stated earlier, Euclid did not refer to ratios as being equal to one another. Similarly, Newton did not refer to any equality in his statement of the law. Accordingly, it is appropriate to refer to (Eq. 1) as the *statement* of a physical law rather than an *equation* because it *does not state that terms are equal*, numerically, or in any other sense.

Clearly, though, physical algebra is now successfully used for used for both applied and theoretical purposes; and modern equations must be compatible with their origins. The chosen example can be used to show this compatibility.

Newton’s Second law is typically stated algebraically as:

(2) F=ma,

where *p* = *m* *a* or, as Newton (1846, p. 72) put it: “The quantity of motion [momentum] is the measure of the same, arising from the velocity and quantity of the matter conjointly.”

To see the parallel between statement (1) and Eq. 2, we can express the terms as ratios between magnitudes and a unit as follows:

(3) $${\text{f}}^{\prime }:\left[\text{f}\right]::{\text{m}}^{\prime }:\left[\text{m}\right]\cdot {\text{a}}^{\prime }:\left[\text{a}\right],$$

where *F* = f′:[f],*m* = m′:[m] and *a* = a′:[a]. Here [f], [m], and [a] are taken to be a unit of force, mass, and acceleration respectively. The term “.” signifies that the two ratios are compounded in the sense of the term used by Euclid in geometry, and later by Galileo (1638) to express complex physical relations. The concept of compounding ratios to express complex ratios will be explained to follow.

Clearly, Eq. 3 and statement (1) share a similar form, with “=” in Eq. 3 substituted for “::” in statement (1) and algebraic multiplication used in Eq. 3 in place of compounding in statement (1). The additional step (which would not have been taken by Euclid) of letting *F* = f′:[f], and so on, equates ratios with numbers. This step is commonly invoked in metrology. Wallis (1670) made precisely this step, from “::” to “=,” although Roche (1998, p. 99) states that “passages elsewhere in Wallis strongly suggest that he interpreted this expression, not as a true algebraic equation, but as an abbreviated ratio equation which related a compound ratio to a simple ratio.”

### Compound ratios in proportionality statements

It appears that the term *compound ratio* is not explicitly defined historically in most sources, other than through reference to specific examples. Where two ratios are compounded, the second quantity of the first ratio is the first quantity of the second ratio, and the compounded ratio is that of the first to the last quantity. The historical roots of compounding are likely in the physics of music, where it is possible to compound two musical intervals to obtain a given ratio (Grattan-Guinness, 1996).

Galileo was able to use compound ratios by making one ratio of lengths proportional to two speeds, and another ratio of lengths proportional to two time intervals. By compounding the two ratios of lengths (first to second with second to third) he demonstrated that the distances of bodies, with different velocities traveled in different time intervals, “bear to each other the compound ratio of the speeds and time intervals” (Galileo, 1638, p. 194). Analogous reasoning can be employed to demonstrate the proportionality in Eq. 3 follows from Newton’s law. The following emulates Galileo’s reasoning.

Consider a body A subjected to a force *f_A_* to accelerate the body at *a_A_*. Consider also a body B subjected to a force *f_B_* to accelerate the body at *a_B_*. The proposition to be demonstrated is that the ratio of *f_A_* to *f_B_* is the compound ratio of masses and accelerations. The proposition can be demonstrated in two stages with reference to Newton’s law.

First, consider the proportionality

(4) $${\text{f}}_{A}:\text{F}::{\text{m}}_{A}:{\text{m}}_{B}.$$

Since the ratio of forces required to accelerate two bodies at the same acceleration is proportional to the ratio of the masses of those bodies, and since *f_A_* moves body A with acceleration *a_A_*, it follows that F is the force required to move body B having mass m_*B*_ with acceleration *a_A_*.

Second, consider also the proportionality

(5) $$\text{F}:{\text{f}}_{B}::{\text{a}}_{A}:{\text{a}}_{B}.$$

Since the ratio of forces required to accelerate a single body at two differing accelerations is proportional to the ratio of those accelerations, and since F is the force required to move body B with acceleration *a_A_*, it follows that f_*B*_ is the force required to move body B with acceleration a_*B*_.

It is thus shown that the proposition of a compound ratio holds. The logic is as follows. Suppose we were to think of the change in force from that required to accelerate body A at *a_A_* to that required to accelerate body B at a_*B*_ through an intermediate stage. In the first stage, using Newton’s terminology, f_*A*_ is *augmented* (or *diminished*) by a particular amount to become F and the alteration of force is such that f_*A*_:F is proportional to m_*A*_:m_*B*_. In the second stage, F is augmented (or diminished) to become f_*B*_ and the alteration of force is such that F:f_*B*_ is proportional to a_*A*_:a_*B*_. That is, the first and last forces stand in the ratio f_*A*_:f_*B*_ and the combination of stages is abbreviated as the compound ratio f_*A*_:F·F:f_*B*_. Compounding thus refers to the relation between an initial and final quantity through their relations with a common or intermediate quantity.

The proportionality of the ratio of forces to the compound ratio can be stated directly as follows:

(6) $${\text{f}}_{A}:{\text{f}}_{B}::{\text{f}}_{A}:\text{F}\cdot \text{F}:{\text{f}}_{B}.$$

The proportionality of the ratio of forces can also be stated “indirectly” in terms of the compound ratio of masses and accelerations, as follows:

(7) $${\text{f}}_{A}:{\text{f}}_{B}::{\text{m}}_{A}:{\text{m}}_{B}\cdot {\text{a}}_{A}:{\text{a}}_{B}.$$

In statement (7), again the force can be thought of as though it changes through an intermediate stage. In the first stage, the alteration of force (from f_*A*_ to f) is proportional to m_*A*_:m_*B*_ and in the second stage the alteration of force (from F to f_*B*_) is proportional to a_*A*_:a_*B*_. The combined alteration is from f_*A*_ to f_*B*_. It is not necessary, of course, that there is an actual process by which the forces change through successive stages. Instead, statement (7) is intended to summarize the complex set of relations among the ratios of forces, masses, and accelerations. In effect, statement (7) is no more or less than an abbreviation for the full line of reasoning used to demonstrate that the proposition holds.

As such, “Euclid’s method of compounding ratios is not at all the same as multiplication, although the two theories exhibit structural similarity” (Grattan-Guinness, 1996, p. 362). The structural similarity is evident by comparing statement (7) with the equation *F* = *m* × *a* given the modern tendency to think of ratios as single numbers. Physical algebra is thus a further abbreviation for the full line of reasoning. Provided a coherent system of units is used there is a direct parallel between proportionality statements and physical algebra, as discussed next.

### Metrological rules for multiplying quantities

It is common in metrology to treat ratios as pure numbers. If the ratios in statement (7) are treated as numbers, multiplication of measurements yields a result that agrees with the law, but only if coherent units are used. A *coherent* system of units is one in which units are defined so as to avoid introducing multiplicative constants that are regarded as “superfluous” (e.g., constants of the kind required to convert imperial to metric). *Provided* that units from a coherent system of units are used, many of the equations of physics can be understood as short-hand symbolic devices for proportionality statements involving compound ratios. In particular, it is possible to understand the equations of physics that are invoked in definitions of SI units this way. However, it is only by virtue of a coherent body of physical relations that a coherent system is possible in the first place (de Boer, 1994/95).

Following from this point, coherent units are defined using these equations in combination with the metrological “rule” that “the value of the product of the values of two concrete quantities, in a given system of measurable quantities and units, is the product of their numerical values and a unit of the new quantity, if such a realizable quantity can exist” (Emerson, 2008, p.136). In the past, the connection between this rule and its historical origins was recognized. As stated by Roche (1998, p. 108): “Throughout the seventeenth and eighteenth centuries the product or division of physical quantities was often understood as an abbreviated statement of a compound ratio.” The example of Newton’s second law of motion therefore illustrates the general connection between modern algebraic expression and the historical origins of physical science.

It would be interesting to consider the ontological and empirical basis for the concept of the proportionality of ratios. However, to do so is well beyond the scope of this paper. It suffices here to emphasize that physical relations were first conceived as proportionalities among ratios and that this conception therefore seems to warrant careful consideration.

## Modern-Day Measurement in Physics

We are now in a position to consider the implications of the historical and conceptual analysis for the way in which measurement is understood today. Direct measurement is considered first because without it, there would be no indirect measurement. Indirect measurement is then considered.

Direct measurement establishes the proportionality between a ratio of continuous quantities and a ratio of discrete quantities. For example, we may establish that the ratio of a length L to unit length [L] is proportional to the ratio of *r* wavelengths to a single wavelength, as expressed in the following proportionality statement:

(8) $$\text{L}:\left[\text{L}\right]::r<\text{wl}>\text{:}<\text{wl}>\text{,}$$

where <wl> refers to wavelength and [L] is a unit of length. [The use of <> to denote a discrete entity is notation adopted by Cooper and Humphry (2012) to be compatible with the convention in metrology of denoting units of continuous quantities using square brackets]. In practice, for instance, it is possible to establish the ratio of a distance to a single wavelength by using an instrument, such as an interferometer, that makes it possible to count the number of wavelengths of light spanning the distance to be measured.

The above example of direct measurement is particularly relevant to the SI because the base units of distance and time are currently defined in terms of wave phenomena, and there is a proposal to define the kilogram in terms of the Planck relation in the future (Mills, 2010). The prototypical examples of direct measurement used by Krantz et al. (1971) involve the formation of a “standard series” as obtained, for example, by concatenating rods of equal length. A standard series can be used to establish a proportionality such as L:[L]::*r*<d>:<d>, where *d* refers to a rod. It is stressed that there must be a clear substantive basis for establishing that ratios of continuous quantities are proportional to ratios of discrete quantities, such as the concatenation of bodies to give a combined mass, or the mixing of volumes of substances with different temperatures in a specific manner to give an intermediate temperature (Kyburg, 1984; Sherry, 2011).

As we have seen, there was long ago a merging of the concept of ratio and number. It is customary now to treat the ratio *r* <wl>:1 <wl> as the number *r*. Following this trend, and using “=” instead of “::,” statement (8) will typically be expressed as the equation L:[L] = *r*, according to which the ratio of lengths is equal to a real number. The classical theory is therefore premised on an abbreviation of the manner in which proportionality of ratios of continuous quantities to ratios of discrete quantities was originally understood.

In metrology, most units within the International System (SI) are defined in a way that involves laws and/or definitions, and without directly involving relations between continuous quantities and discrete quantities. Measurement approached in this manner was referred to by Kyburg (1984) as indirect and/or systematic measurement and is effectively what Campbell (1928) referred to as derived measurement.

Indirect measurement is exemplified by the definition of the unit of force. Maxwell (1876, art. SLVII) said that “the unit of force is that force which, acting on the unit of mass for the unit of time, generates the unit of velocity.” The SI unit of force, the newton, is also defined in precisely this way. The definition is based on Newton’s second law of motion, which can be expressed in terms of SI units using the following proportionality statement:

(9) $${\text{f}}^{\prime }:\text{N}::{\text{m}}^{\prime }:\text{kg}\cdot {\text{a}}^{\prime }:\text{a},$$

where N is the newton, kg is the kilogram and a is the unit of acceleration, 1 m per second, per second. Using indirect measurement, it is possible to establish a ratio between a magnitude, such as F′, and a unit, such as the newton, by using an instrument and procedure designed for the purpose. Any such instrument must be designed on the basis of physical relations that include, but are not limited to, that relation used to indirectly define the unit. In the chosen example, the instrument will typically be designed such that a force acts on a mass to accelerate it under controlled conditions.

## Implications for the Social Sciences

A detailed analysis of the possible implications for the social sciences is beyond the scope of this paper. Nevertheless, it is possible to touch on some pertinent considerations and to make some preliminary observations.

One obvious question that might be asked is whether it is possible to understand psychological phenomena in terms of proportionality and ratio analogous to the manner in which physical relations are understood. Do psychological attributes exist that are proportional to one another under specific conditions? Do psychological attributes exist that are proportional to physical attributes under specific conditions? If the answer to either question were yes, it could provide a basis for the measurement of psychological attributes through indirect measurement.

It may be more fertile to begin, however, by asking a rather different question: are there psychological faculties for recognizing ratios and proportionality? It seems that such faculties must exist, for physical ratios are grasped by perception through acquaintance with physical phenomena. It is not likely that human beings could perceive ratios as a basic kind of relation, and successfully develop physics on this basis, without any sort of sensory and mental apparatus for doing so.

A simple example showing humans likely possess such a faculty is the horizon-ratio relation, which applies to vertically extended objects such as trees and poles. The horizon-ratio is the ratio of the proportion of the object’s height seen above the horizon to the proportion of the object’s height seen below the horizon. Irrespective of the distance from an observer, this ratio is the same for any object of the same height (Sedgwick, 1973; Gibson, 1986; Rookes and Willson, 2000). Thus, for example, if a series of light poles of equal height extends toward the horizon, an observer will see the same ratio of the proportion of height above to below the horizon for each pole. There is evidence that people do draw upon this relation, whether the actual horizon or an implicit horizon is used as the reference (Bermanini et al., 1998). The more general prediction that can be made is that humans directly perceive and respond to ratios, and proportionalities of ratios, in ways that are relevant to functional and goal-oriented actions and responses. This would be significant for psychophysics because it means focusing on how humans perceive physical relations involved in functional behavior as much as focusing on sensory responses to stimuli, which has been the major focus of the discipline through its history.

Although there is a complex divergence of views, historically psychophysics has focused in particular on posited relationships between *physical stimuli* and *sensations*, where the latter are taken to be quantitative attributes. It was observed some time ago, however, that a human capacity to directly perceive and estimate ratios is a parsimonious explanation for empirical results in psychophysics, as follows.

Why must we suppose that [a person] bases his estimates of physical ratios on estimates of psychological ratios? Why is the following explanation not sufficient? [The person’s] eyes are normal and he has learned how rods look when they stand in the ratio 1:2; hence when he looks at rods he can say with some accuracy whether or not they stand in that ratio (Savage, 1970, p. 383).

If humans possess faculties by which to perceive and functionally respond to physical ratios, another possible avenue becomes available. It may be fruitful to study sensory and perceptual apparatus much as we might study a complex set of measuring instruments. Such an approach essentially occurred during the course of early endeavors to measure the intensity of light. In that context, a “central problem concerned the basing of standards of brightness on highly variable human observers, and on the complex mechanism of visual perception.” (Johnston, 2001, p. 7) Indeed, much existing psychophysics can be understood from this perspective, without needing to refer either to sensory magnitudes or the representation of psychological attributes by numbers. It would, however, seem preferable to consider ways in which estimates are implied by functional responses, for there is no reason humans should have evolved the ability to verbally report ratios of any given kind of quantity under a given set of conditions.

### Direct and indirect measurement in the social sciences

The question considered earlier is whether it is possible to understand psychological phenomena in terms of proportionality and ratio. To be consistent with the origins of quantitative science, the challenge is to either directly or indirectly measure a posited quantitative psychological attribute by establishing such proportionalities.

The possibility or otherwise of direct measurement in psychology has been debated for some time, perhaps most notably as part of the deliberations of the Ferguson Committee (Ferguson et al., 1940). Stevens attempted to circumvent these implications. Krantz et al. (1971) sought to provide an axiomatic basis for measurement that avoids the need for empirical concatenation operations. The axiomatic representational approach is premised on the idea that a theory of measurement is required to successfully measure an attribute in psychology. History shows, however, that physics did not progress based on application of representational measurement theory. The historical analysis in this paper indicates that *physical* theory, originally expressed in terms of proportionality and ratio, forms the foundation for measurement. Consistent with this, it is clearly evident from applied metrology that substantive theory is used to design instruments and procedures (Hebra, 2010, p. 7). This dependence of measurement in physics on substantive theory is also explicitly reflected in the definitions of units in the SI (de Boer, 1994/95; Massey, 1971; Emerson, 2004; Humphry, 2011a). Thus, Krantz et al. (1971) advocated an approach to measurement in the social sciences that differs from the approach taken in physics in that they invoked “theories different from those that have worked in physics” (p. 17).

Although the representational theorists allow that the measurement of some quantities can be based on physical law, Krantz (1972) claimed that the “measurement of physical quantities such as length, mass, and duration is logically prior to the formulation of the quantitative laws of physics.” It has been seen, however, that the SI units of length and duration are defined in terms of physical theory and law, and that there is a proposal to define the unit of mass in terms of a physical relation. The definitions of SI units are based on physical quantitative relations involving the relevant kinds of quantities, consistent with the historical mode of expressions of relations in physical science. These definitions have a related *mise en pratique* (a set of applied instructions) for measuring in the units based on the definitions. This means it is possible to approach the measurement of these quantities by using theory, definition, and law to design measuring instruments and procedures. Even putting aside the question of whether the measurement of quantities such as mass can be approached in this fashion, the measurement of length can be achieved based on wave phenomena without recourse to concatenation operations. Length measurement was used as the prototypical case in setting up the foundations. It is therefore by no means clear whether the measurement of physical quantities such as length based on concatenation operations is logically prior to the formulation of quantitative laws. Instead, it remains possible that the measurement of most, if not all, physical quantities can be approached on the basis of understanding physical relations.

Thus, it has been the case that in attempts to connect psychology to fundamental measurement as defined by Campbell (1928, p. 14), direct measurement based on concatenation or an analogous operation has received the most attention (without success, at least to date). Much less attention has been given, at least explicitly, to what Kyburg (1984, p. 143) referred to as “systematic measurement” as a primary mode of approaching measurement. Systematic measurement involves quantities that are systematically related within a theoretical framework. It would be somewhat surprising if a quantitative attribute existed in psychology that did not have a relationship with any physical attribute. It seems more likely instead that quantitative psychological attributes would be related to (or an extension of) physical quantities, which would make it reasonable to refer, as Berka (1983, p. 13) did, to “extraphysical measurement.” To the extent that this assumption is correct, systematic, and indirect measurement would seem more likely to succeed.

It is simple enough to posit quantitative relations in the social sciences that are analogous in purely formal terms with those that form the basis for measurement in physics (see Humphry, 2011b). Typically, though, one of a few problems exists. In some cases, posited relations involve only continuous quantities and there is no footing in direct measurement. In other cases, discrete quantities are involved but there is no clear and transparent explanation for the proportionality of ratios of continuous quantities and ratios of discrete quantities (although possible exceptions exist in Psychophysics and Economics). In other cases still, exponential and logarithmic relations are invoked in a manner that entails a disjunction with the origins of physics. The challenge is to ascertain whether there is a productive approach to systematic measurement that avoids such problems.

## Summary and Conclusion

It has been seen that following the Greek-inspired tradition, pioneers of physics such as Galileo, Newton, Coulomb, and Faraday explicitly stated physical relations as proportionalities among ratios. In this tradition, ratios were seen as being in the category of relation. Because it centers on this concept of ratio, the classical definition of measurement is more congruent with the origins of quantitative sciences than the representational theory. During a certain period of history, though, numbers were used to represent ratios and lines and eventually it became commonplace to interpret symbols in physical equations as numbers. Algebra emerged as a short-hand abbreviation of proportionalities among ratios. These and other developments paved the way for the emergence of the representational view of measurement.

A compact form of notation was used to show the parallel between the Greek-inspired tradition and modern algebra. This parallel is seemingly the reason that the classical view of measurement continues to exist alongside the representational view in physical sciences. However, in the social sciences, representational definitions predominate. The likely reason for this is the failure to establish a basis for measurement that is directly connected to its origins in physical science.

Understood in light of the origins of physico-mathematics, the objective of measurement is to establish that quantities stand in a specific ratio to one another. This objective can be approached by a combination of direct and indirect measurement. If something akin to indirect measurement is invoked, a basic challenge for the social sciences is to establish a clear footing in direct measurement. A more immediate avenue to approaching measurement in the social sciences is to advance our understandings of the ways in which humans perceive and functionally respond to ratios and proportionalities among ratios.

## Conflict of Interest Statement

The author declares that the research was conducted in the absence of any commercial or financial relationships that could be construed as a potential conflict of interest.
